# MHC II-PI_3_K/Akt/mTOR Signaling Pathway Regulates Intestinal Immune Response Induced by Soy Glycinin in Hybrid Grouper: Protective Effects of Sodium Butyrate

**DOI:** 10.3389/fimmu.2020.615980

**Published:** 2021-01-18

**Authors:** Bin Yin, Hongyu Liu, Beiping Tan, Xiaohui Dong, Shuyan Chi, Qihui Yang, Shuang Zhang

**Affiliations:** ^1^ Laboratory of Aquatic Animal Nutrition and Feed, Fisheries College, Guangdong Ocean University, Zhanjiang, China; ^2^ Aquatic Animals Precision Nutrition and High Efficiency Feed Engineering Research Centre of Guangdong Province, Zhanjiang, China; ^3^ Key Laboratory of Aquatic, Livestock and Poultry Feed Science and Technology in South China, Ministry of Agriculture, Zhanjiang, China

**Keywords:** hybrid grouper (*Epinephelus fuscoguttatus*♀×*E. lanceolatus*♂), soy glycinin, sodium butyrate, intestinal inflammation, MHC II-PI_3_K/Akt/mTOR signaling pathway

## Abstract

Soy glycinin (11S) is involved in immune regulation. As an additive, sodium butyrate (SB) can relieve inflammation caused by 11S. To further delve into the mechanisms. A diet containing 50% fishmeal was the control group (FM group), and the experimental groups consisted of the FM group baseline plus 2% glycinin (GL group), 8% glycinin (GH group), and 8% glycinin + 0.13% sodium butyrate (GH-SB group). The specific growth ratio (SGR), feed utilization, and density of distal intestinal (DI) type II mucous cells were increased in the GL group. In the serum, IFN-γ was significantly upregulated in the GL group, and IgG and IL-1β were upregulated in the GH group. IgG, IL-1β, and TNF-α in the GH-SB group were significantly downregulated compared to those in the GH group. The mRNA levels of mTOR C1, mTOR C2, and Deptor were upregulated in the GL, GH, and GH-SB groups in the DI compared with those in the FM group, while the mRNA levels of mTOR C1 and Deptor in the GH group were higher than those in the GL and GH-SB groups. 4E-BP1, RICTOR, PRR5, MHC II, and CD4 were upregulated in the GH group. TSC1, mLST8, and NFY mRNA levels in the GL and GH-SB groups were upregulated compared with those in the FM and GH groups. Western blotting showed P-PI_3_K^Ser294^/T-PI_3_K, P-Akt^Ser473^/T-Akt, and P-mTOR^Ser2448^/T-mTOR were upregulated in the GH group. Collectively, our results demonstrate that low-dose 11S could improve serum immune by secreting IFN-γ. The overexpression of IgG and IL-1β is the reason that high-dose 11S reduces serum immune function, and supplementing SB can suppress this overexpression. Low-dose 11S can block the relationship between PI_3_K and mTOR C2. It can also inhibit the expression of 4E-BP1 through mTOR C1. High-dose 11S upregulates 4E-BP2 through mTOR C1, aggravating intestinal inflammation. SB could relieve inflammation by blocking PI_3_K/mTOR C2 and inhibiting 4E-BP2. Generally speaking, the hybrid grouper obtained different serum and DI immune responses under different doses of 11S, and these responses were ultimately manifested in growth performance. SB can effectively enhance serum immunity and relieve intestinal inflammation caused by high dose 11S.

## Introduction

Hybrid grouper (*Epinephelus fuscoguttatus*♀×*E. lanceolatus*♂) is a type of broad salt tolerant fish in warm coastal waters. It is widely cultivated in southern China. Characteristics of hybrid groupers include fast growth and strong disease resistance. In 2017, the Chinese Ministry of Agriculture included groupers into the national marine fish industry technology system. As a carnivorous marine fish, hybrid grouper feed typically is up to 50% protein, with a content of fish meal also as high as 50%. The addition of a large amount of fish meal to the feed during the breeding period is the main cause of high phosphorus and nitrogen pollution in aquaculture water. Nitrogen and phosphorus are the two main pollutants in aquatic environments (1). Fish meal usually contains 51.1–73.0% crude protein and 1.67–4.21% phosphorus, whereas plant-based protein, such as soybean meal, usually contains 44.8–50% crude protein and only 0.6–0.7% phosphorus (2). Therefore, the use of plant-based protein to replace some of the fish meal in fish feed can reduce the feed’s crude protein and phosphorus content, thereby reducing nitrogen and phosphorus pollution in the breeding water. To avoid excessive pollution, scholars have long committed to using plant proteins to replace fish meal. Soybean meal, a common source of plant protein, is widely available at low prices. However, it contains a large amount of anti-nutritional factors such as glycinin and β-conglycinin, which cause groupers to have a very low tolerance for it.

Glycinin (11S) is one of the most immunogenic factors in soybean antigen protein, accounting for about 40% of the total protein content in soybeans. Its thermal stability leads to its weak inactivation ability under normal heating treatment. Too much glycinin may cause intestinal allergies and disrupt the intestinal barrier, which could cause a large amount of nutrients in the feed to be excreted and enter the water instead of being absorbed by the intestine. Unlike in mammals, the antigen-binding epitopes of fish intestines are mainly concentrated in the distal intestine (DI) (3). Differentiated DI epithelial cells are the most susceptible to antigen binding, causing the allergic response to be most pronounced in the DI (4). However, in some experiments in which soybean meal replaced fish meal, the rate of weight gain was improved with low substitution levels (5). We suspect that this phenomenon is related to low-dose 11S immune enhancement. Nevertheless, the hybrid grouper has poor tolerance to soybean meal (6), largely because of its high 11S content (7). Poor growth is mainly due to inflammation in the intestinal tract, which leads to a decrease in the efficiency of nutrient absorption (8). Studies have shown that glycinin causes intestinal allergies in animals and induces intestinal cellular oxidative damage by decreasing antioxidant enzyme activities in juvenile turbot (*Scophthalmus maximus* L.) (9).

The antigen protein 11S enters the body and becomes an allergen with antigenic activity, stimulating allergic reactions in the intestinal mucosal immune system. This causes intestinal damage, intestinal permeability changes, digestion issues, and malabsorption. There are four types of allergic reactions: Type I is an acute allergic reaction mediated by the specific antibody IgE; Type II is cytotoxic response. Cytolysis or tissue damage due to complement involvement when an antigenic antibody reaction with the corresponding antigen occurs. Type III is a delayed reaction mediated by a specific antigen-antibody complex; and Type IV is a delayed allergic reaction mediated by specific T lymphocytes (10). However, the exact mechanisms of intestinal allergy type and inflammatory response induced by 11S in carnivorous marine fish have not been reported.

Sodium butyrate (SB) has been widely used in livestock and poultry as an alternative to antibiotics. It has also been applied to aquatic animals in recent years and has achieved good results (11). Its active ingredient, butyric acid, provides energy directly to intestinal epithelial cells without being absorbed by the hepatobiliary system or entering the tricarboxylate transport system. It maintains the normal state of intestinal mucosal epithelial cells and promotes digestion and absorption of the small intestine (12). SB may increase the antioxidant capacity of grass carp by inhibiting apoptosis-related products and improving the integrity of intestinal cell structure by upregulating intestinal zonula occludens-1 (ZO-1), zonula occludens-2 (ZO-2), and claudin-b protein (13). In addition, SB can relieve inflammation by anti-oxidation. It can reduce xanthine oxidase activity in the intestinal mucosa of rats with ulcerative colitis, as well as reduce glutathione content, oxygen free radicals, and lipid peroxidation of unsaturated fatty acids in cells. However, no systematic study has been conducted on the repair effect of SB on intestinal abnormalities caused by antigen protein 11S in hybrid groupers. Presently, there are no studies on the application of SB to hybrid groupers. In this experiment, the immune regulatory effect of 11S on the hybrid grouper was investigated. To provide a theoretical reference for improving the tolerance of hybrid grouper to soybean meal protein sources, supplementation with SB was used to repair the intestinal inflammation caused by the high level of antigenic protein 11S.

In this study, the hybrid grouper, an economic fish widely farmed in southern China, was chosen as the subject of our experiment. We were interested in comparing the differences between different levels of glycinin and SB repair effects. We wanted to test the effects of glycinin and SB on growth performance, serum biochemical indices, distal intestinal morphology, and inflammation.

## Material and Methods

### Animals

The fish used in this experiment were purchased from a commercial hatchery in East Island (Zhanjiang, China), temporarily cultured in a cement pond at the Biological Research Base of Guangdong Ocean University, and domesticated with commercial feed for 7 days. The animal protocol was approved by the ethics review board of Guangdong Ocean University. All procedures were performed according to the standards of the National Institutes of Health Guide for the Care and Use of Laboratory Animals (NIH Publication No. 8023, revised 1978) and relevant Chinese policies.

### Feeding Trial and Challenge Test

After they were stable, 480 healthy experimental fish of the same weight were randomly selected and allocated to 16 plastic barrels reinforced with glass fiber, with 30 fish per barrel. The fish were cultured for 8 weeks. The fish were separated into four treatment groups. Every day, each treatment was repeated 4 times, full feeding was performed at 8:00 and 17:00, and 70% of the water was replaced. During the experiment, the water temperature was 29.00 ± 1.30°C, salinity was 34.00 ± 2.00 ‰, dissolved oxygen concentration was ≥7.00 mg/L, pH was 7.80–8.10, and ammonia nitrogen concentration was ≤0.09 mg/L. At the end of the 8-week feeding trial, 40 fish were randomly selected from each group (10 fish per replicate). Following the methodology by Yin et al. (5), each fish was intraperitoneally injected with 200 μl of *Vibrio parahaemolyticus* at a concentration of 7.41 × 10^8^ CFU/ml and observed for a week until stabilized. The cumulative mortality of each group was counted.

### Diet Formulations

The control groups (FM group) were fed with a control diet (0% glycinin, 0% SB). The experimental groups (named GL, GH, and GH-SB, respectively) were fed with diet GL (2% glycinin, 0% SB), diet GH (8% glycinin, 0% SB), and diet GH-SB (8% glycinin, 1.33% SB). Methionine and lysine were supplemented in GL, GH, and GH-SB diets. Nutrient composition and amino acid profiles are shown in **Tables S1** and **S2**, respectively. Purified glycinin (from soybean meal) was purchased from China Agricultural University (patent no. 200410029589.4, China). Water was added to adjust the pH of the 11S suspension to 7.2. The suspension was freeze dried for 72 h to make the 11S solid, and a small pulverizer was used to crush it. Microencapsulated SB (30%) was kindly provided by Shanghai Menon Animal Nutrition Technology Co., Ltd. All feed ingredients were crushed into powder through a 380 μm mesh. After all the ingredients were combined, fish oil, soy lecithin, and water were added and mixed thoroughly. After being pelletized and air dried for 2–3 days at room temperature, all the feed was kept in a refrigerator (-20°C) until use.

### Sample Collection and Analysis

After 8 weeks of the feeding trial, the fish were starved for 24 h before being weighed and counted. Three fish from each tank were randomly selected to have their blood drawn using a 1 mL needle tube. The blood samples were placed in a 1.5-ml centrifuge tube on ice for temporary storage and then put in a 4°C refrigerator to rest. The samples were centrifuged at 3500 rpm for 15 min. The supernatant was carefully separated and stored at -80°C to be later used for measuring enzyme activity and testing related biochemical indicators. Three new fish were randomly selected, and the DI from each fish, from the anus to the first corner of the intestine, was removed. After removing it, it was quickly washed in PBS, and water was absorbed on qualitative filter paper. One of the DIs was stored in a 10-ml centrifuge tube containing 5 ml of 4% paraformaldehyde for AB-PAS section preparation. The other two DIs were temporarily stored in liquid nitrogen and placed in a -80 °C refrigerator to be used for determination of gene and protein expression.

### Distal Intestinal Alcian Blue-Periodic Acid Schiff (AB-PAS) Section

For histopathological analyses, tissues were first embedded in paraffin, cut into 3–4 μm thick sections, and AB-PAS stained as described by Bergström et al. (14). A Leica DM 6000 optical microscope was used to observe 10 randomly selected plicas and muscle thicknesses in each section. The number of type II mucous cells on each plica was measured using the cellSens Standard 1.8 software, and the number of type II cells per millimeter was calculated.

### Total RNA Extraction and cDNA Synthesis

Total RNA extraction was performed on 100–150 mg of the distal intestine using 1 ml of Trizol (TRI Reagent solution, Invitrogen, Carlsbad, CA, USA) according to the instructions previously described by Orriss et al. (15). The RNA quality and quantity were assessed by electrophoresis on 1% agarose gels and spectrophotometric analysis with NanoDrop 2000 (A260:280 nm ratio), respectively. The PrimerScript™ RT-PCR Kit (TaKaRa, Kusatsu, Japan) was used to reverse-transcribe RNA into cDNA according to the manufacturer’s instructions. Specific primers (**Table S3**) were designed according to the full-length sequences from transcriptome sequencing (not published) of the hybrid grouper. An Applied Biosystems 7500 Real-Time PCR System (Life Technologies, Carlsbad, CA, USA) was used to perform all the real-time PCR reactions using a SYBR ^®^ Premix Ex Taq™ Kit (Takara). Relative gene expression was analyzed using the 2^-ΔΔCT^ method according to Livak et al. (16).

### Total Protein Extraction and Sodium Dodecyl Sulfate-Polyacrylamide Gel Electrophoresis (SDS-PAGE)

PBS (1x), cell lysate, protease inhibitors, phosphorylase inhibitors, and PMSF were added to intestinal tissues for low-temperature fragmentation and centrifuged at 12,000 rpm for 20 min. Then, the intermediate layer was collected, and the protein concentration was determined using the Beyotime BCA kit using bovine serum as a standard, as described by Xing et al. (17). According to the experimental requirements of the Western Blot, loading buffer and PBS were added to the protein sample to make the final concentration 2 μg/μl. The EP tube containing the sample was then placed in boiling water for 10 min, quickly put on ice, and then moved to a -80°C refrigerator until use. SDS-PAGE was used to separate the protein samples (20 μg total protein per glue hole). The gel was run with a 90 V electrophoresis instrument for about 30 min, transferred to a 0.45 μm PVDF membrane (Millipore), and run under 110 V for approximately 80 min. The membrane was blocked with 5% skim milk powder in TBST buffer (band) for 1 h at room temperature. TBST buffer was used to wash the membrane three times for 10 min each time. The primary antibody was incubated at room temperature for 2 h, washed three times with TBST for 10 min each time, and then incubated with the secondary antibody at room temperature for 1 h. ECL reagents (Millipore) were then used to visualize the membrane (band). The following antibodies were used in this study: antibodies against PI_3_ kinase p85 (4292), phospho-PI_3_ kinase class III (Ser^249^, 13857S), Akt (9272S), phospho-Akt (Ser^473^, 9271S), mTOR (2972S), phospho-mTOR (Ser^2448^, 2971S), and GAPDH (2118S). All antibodies were purchased from Cell Signaling Technology. Since mammalian antibodies were used, amino acid sequences of the studied protein from hybrid grouper were aligned in the NCBI database (https://blast.ncbi.nlm.nih.gov/Blast.cgi) to check the identity of the antibodies. Western bands were quantified using NIH Image 1.63 software.

### Calculations and Statistical Analysis

WGR (weight gain rate, %) = 100 × (final weight - initial weight)/initial weight

SGR (specific growth rate, %/day) = 100 × (ln(final weight) - ln(initial weight))/days of experiment

SR (survival rate, %) = 100 × final fish number/initial fish number

FCR (feed coefficient ratio) = feed consumed/weight gain

Id/Ph = intestinal diameter/plica height

All statistical evaluations were subjected to one-way analysis of variance followed by Tukey’s multiple range tests to determine significant differences among treatment groups. Analyses were done using Social Sciences version 22 (SPSS Inc., Chicago, IL, USA) at a level of *P* < 0.05, as described by Guo et al. (18). The results are presented as the mean ± SE.

## Results

### Growth Performance and Challenge Test

The growth parameters and feed utilization are listed in **Table 1**. The initial body weight of fish in each group was 7.71 ± 0.04 g. After an 8-week feeding trial, fish fed the GL diet gained more SGR than those fed the FM, GH, and GH-SB diets (*P* < 0.05), and no significant difference was observed between the FM and GH-SB groups (*P* > 0.05). Compared with the FM group, the GH group had decreased FCR (*P* < 0.05). After a 7-day challenge test, the cumulative mortality (CM) in the GH and GH-SB groups increased (*P* < 0.05). No significant difference was found for SR (*P* < 0.05).

**Table 1:** Growth parameters and feed utilization of juvenile hybrid grouper (*Epinephelus fuscoguttatus*♀*×E. lanceolatus*♂) fed the experimental diets for 8 weeks.

	FM	GL	GH	GH-SB
IBW (g)	7.70±0.05	7.71±0.03	7.70±0.03	7.72±0.04
FBW (g)	50.60±0.26^b^	58.80±0.54^c^	45.09±0.70^a^	52.29±1.31^b^
WGR (%)	557.22±3.33^b^	663.60±6.95^c^	485.57±9.15^a^	579.14±17.06^b^
SGR (%/day)	3.36±0.01^b^	3.63±0.02^c^	3.15±0.05^a^	3.42±0.08^b^
SR (%)	100.00±0.00	95.83±1.60	99.17±0.83	97.78±2.22
FCR	0.81±0.05^ab^	0.65±0.11^a^	1.35±0.17^c^	0.99±0.06^b^

Value show means ± SE (n = 4); Significance was evaluated by one-way ANOVA followed by Tukey’s multiple range tests. FM, control diet; GL, containing 2% 11S diet, GH, containing 8% 11S diet, GH-SB, containing 8% 11S and 0.13% SB diet. IBW, initial body weight; FBW, final body weight; WGR, weight gain rate; SGR, specific growth rate; SR, survival rate; FCR, feed coefficient ratio. ^a, b, c^Mean values among all treatments with different letters were significantly different when the interaction was significant (P < 0.05).

### Serum Biochemical Indexes

**Figure 1:**
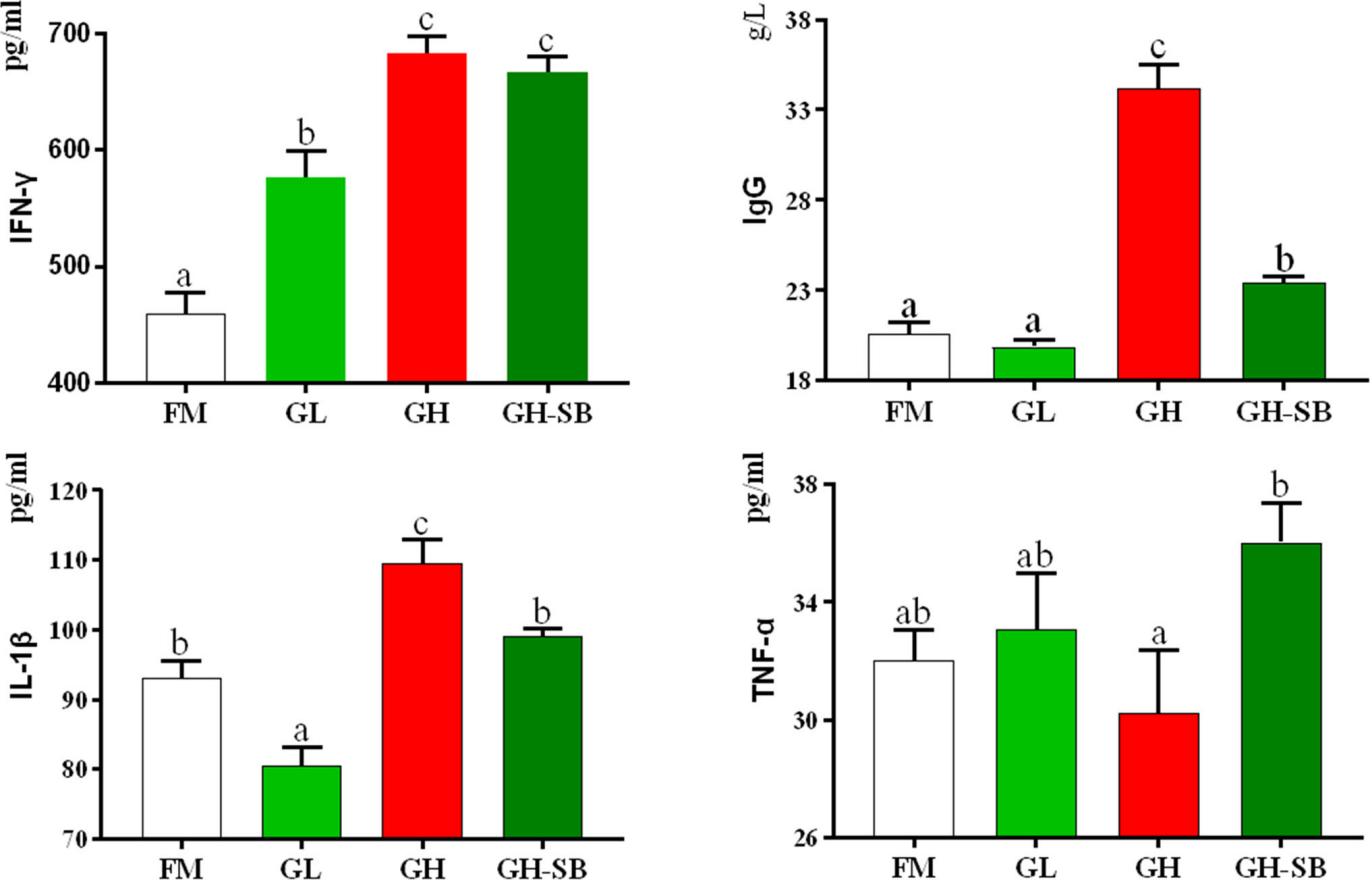
Serum biochemical indexes of juvenile hybrid grouper (*Epinephelus fuscoguttatus*♀*×E. lanceolatus*♂) fed the experimental diets for 8 weeks. Value show means ± SE (n = 4); Significance was evaluated by one-way ANOVA followed by Tukey’s multiple range tests. FM, control diet; GL, containing 2% 11S diet, GH, containing 8% 11S diet, GH-SB, containing 8% 11S and 0.13% SB diet. IFN-γ, interferon-gamma; IgG, immunoglobulin G; IL-1β, interleukin-1 beta; TNF-α, tumor necrosis factor-alpha. ^a,b,c^Mean values among all treatments with different letters were significantly different when the interaction was significant (*P* < 0.05).

The serum biochemical indexes are shown in **Figure 1**. The IFN-γ content in the GL, GH, and GH-SB groups was significantly higher than that in the FM group (*P* < 0.05). IgG levels in the GH and GH-SB groups were significantly higher than those in the FM group (*P* < 0.05). The IL-1β content decreased and increased in the GL and GH groups, respectively, compared with that in the FM group (*P* < 0.05). The TNF-α content in the GH group was significantly higher than that in the GH-SB group (*P* < 0.05), and there was no significant difference among the FM, GL, and GH-SB groups (*P* > 0.05).

### Distal Intestinal Morphological Development

**Figure 2:**
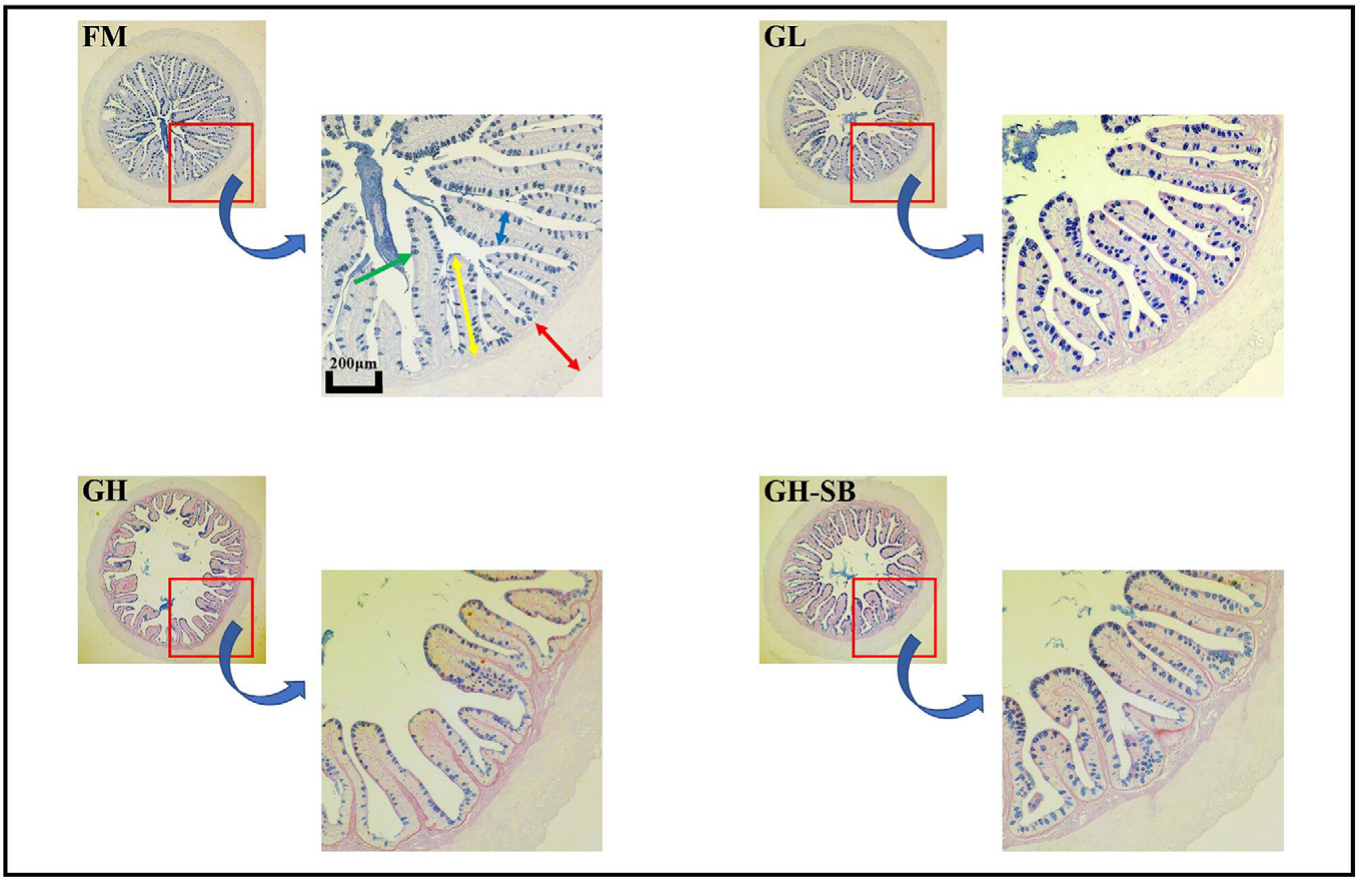
Distal intestinal AB-PAS staining section of juvenile hybrid grouper (*Epinephelus fuscoguttatus*♀×*E. lanceolatus*♂) fed the experimental diets for 8 weeks. Green arrow: type II mucous cell; red arrow: muscle thickness; yellow arrow: plica height: blue arrow: plica width.

**Figure 3:**
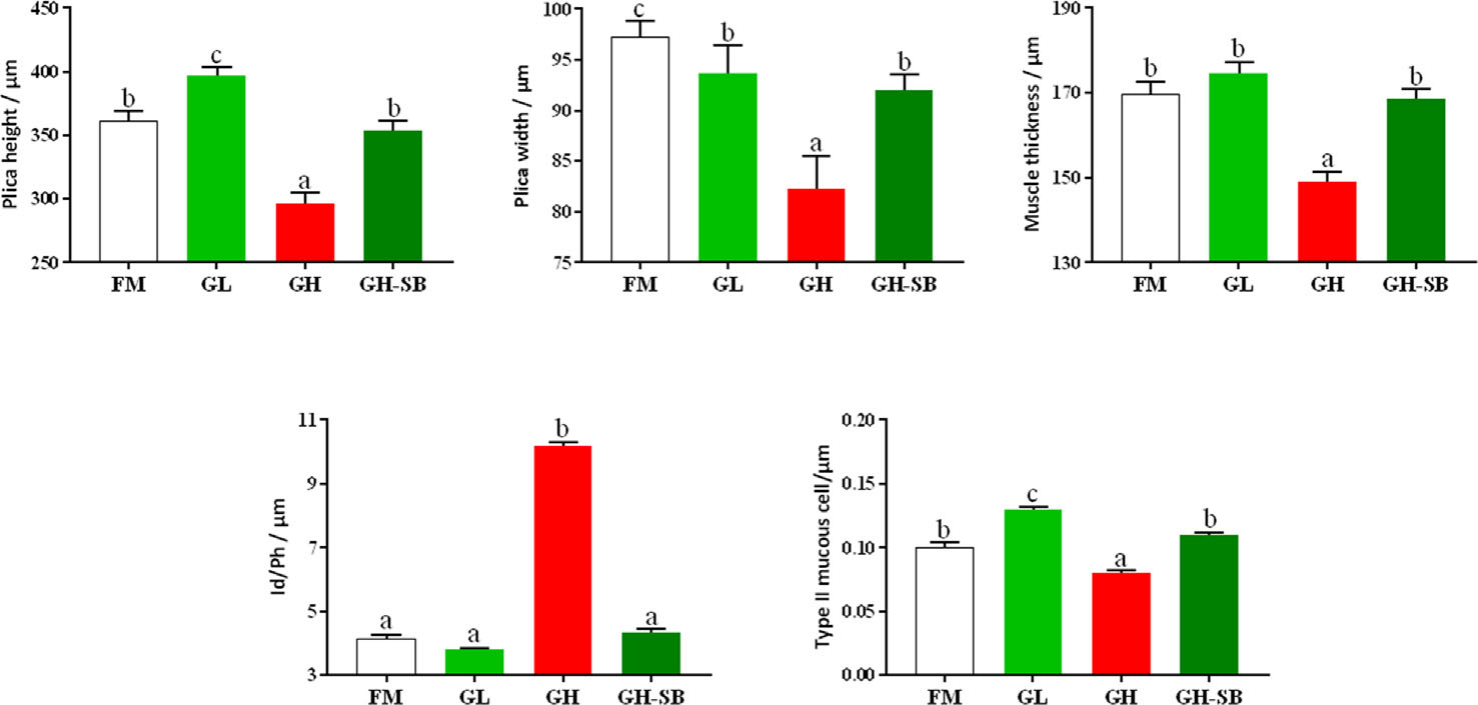
Distal intestinal morphological development of juvenile hybrid grouper (*Epinephelus fuscoguttatus*♀*×E. lanceolatus*♂) fed the experimental diets for 8 weeks. Value show means ± SE (n = 4); Significance was evaluated by one-way ANOVA followed by Tukey’s multiple range tests. FM, control diet; GL, containing 2% 11S diet, GH, containing 8% 11S diet, GH-SB, containing 8% 11S and 0.13% SB diet. ^a,b,c^Mean values among all treatments with different letters were significantly different when the interaction was significant (*P* < 0.05).

The distal intestinal morphological development is shown in **Figure 2**. The plica height, plica width, and muscle thickness (**Figure 3**) in the FM, GL, and GH-SB groups were higher than those in the GH group (*P* < 0.05). The plica height in the GL group was higher than that in the FM group (*P* < 0.05). Id/Ph decreased in the GL group (*P* > 0.05) and increased in the GH group (*P* < 0.05) compared with that in the FM group; there was no difference between the FM group and GH-SB group (*P* > 0.05). The number of type II mucous cells in the GL group was significantly higher than that in the FM and GH-SB groups (*P* < 0.05); this number was significantly lower in the GH group than in the FM, GL, and GH-SB groups (*P* < 0.05).

### Distal Intestinal Immune-Related Genes Expression

**Figure 4:**
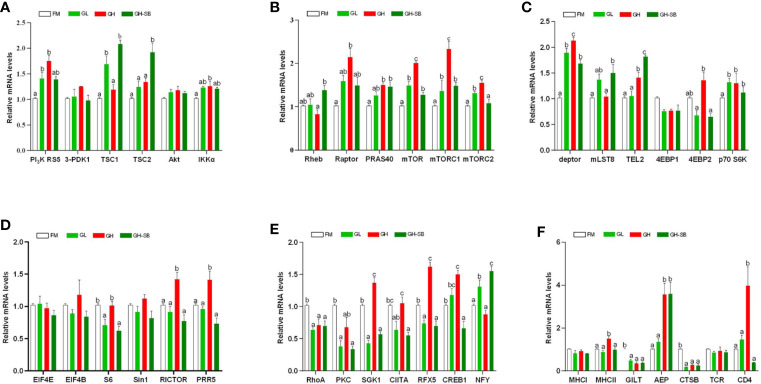
Distal intestinal antigen processing and presentation-related genes expression of juvenile hybrid grouper (*Epinephelus fuscoguttatus♀*×*E. lanceolatus*♂) fed the experimental diets for 8 weeks. Results are represented as mean ± SE (n = 4). **(A)** PI_3_K RS5, phosphatidylinositol 3-kinase regulatory subunit 5; 3-PDK1, 3-phosphoinositide dependent kinase-1; TSC1, tuberous 1; TSC2, tuberous 2; Akt, serine/threonine-protein kinase; IKKα, inhibitor of nuclear factor kappa-B kinase subunit α; **(B)** Rheb, Ras homolog enriched in brain; Raptor, regulatory associated protein of mTOR; PRAS40, proline-rich Akt1 substrate 1; mTOR, mammalian target of rapamycin; mTOR C1, mammalian target of rapamycin complex 1; mTOR C2, mammalian target of rapamycin complex 2; **(C)** deptor, DEP domain-containing mTOR-interacting protein; mLST8, target of rapamycin complex subunit lst8; TEL2, telomere length regulation protein; 4EBP1, eukaryotic translation initiation factor 4E binding protein 1; 4EBP2, eukaryotic translation initiation factor 4E binding protein 2; p70 S6K, ribosomal protein S6 kinase β1; **(D)** EIF4E, translation initiation factor 4E; EIF4B, translation initiation factor 4B; S6, small subunit ribosomal protein S6; Sin1, target of rapamycin complex 2 subunit; RICTOR, rapamycin-insensitive companion of mTOR; PRR5, proline-rich protein 5; **(E)** RhoA, Ras homolog gene family, member A; PKCα, protein kinase C α; SGK1, serum/glucocorticoid-regulated kinase 1; CIITA, major histocompatibility complex class II trans-activator; RFX5, regulatory factor X5; CREB1, cyclic AMP-responsive element-binding protein 1; NFY, nuclear transcription factor Y subunit α; **(F)** MHC I, major histocompatibility complex class I antigen; MHC II, major histocompatibility complex class II antigen; GILT, gamma-interferon-inducible-lysosomal thiol reductase; AEP, asparaginyl endopeptidase; CTSB, cathepsin B; TCR, T cell receptor; CD4, T-cell surface glycoprotein. Significance was evaluated by one-way ANOVA followed by Tukey’s multiple range tests. FM, control diet; GL, containing 2% 11S diet, GH, containing 8% 11S diet, GH-SB, containing 8% 11S and 0.13% SB diet. ^a,b,c^Mean values among all treatments with different letters were significantly different when the interaction was significant (*P* < 0.05).

The distal intestinal antigen processing and presentation-related signaling molecule gene expression in the DI are presented in **Figure 4**. The mRNA levels of RICTOR, PRR5, MHC II, and CD4 were upregulated in the GH group (*P* < 0.05). The mRNA levels of mTOR, mTOR C1, and Deptor were upregulated in the GL, GH, and GH-SB groups compared with those in the FM group (*P* < 0.05). Meanwhile, the mRNA levels of mTOR, mTOR C1, and Deptor in the GH group were higher than those in the GL and GH-SB groups (*P* < 0.05). RhoA, PKC, GILT, and CTSB mRNA levels were downregulated in the GL, GH, and GH-SB groups (*P* < 0.05). The mRNA levels of PI_3_K RS5, IKKα, RAPTOR, PRAS40, mTOR C2, TEL2, p70 S6K, AEP, SGK1, CIITA, RFX5, CREB1, and 4EBP1 were upregulated in the GH group compared with those in the FM group (*P* < 0.05), and SGK1, CIITA, RFX5, CREB1, and 4EBP1 were downregulated in the GH-SB group (*P* < 0.05). TSC1, mLST8, and NFY mRNA levels in the GL and GH-SB groups were upregulated compared with those in the FM and GH groups (*P* < 0.05). The mRNA levels of TSC2 and Rheb in the GH-SB group were upregulated compared with those in the GH group (*P* < 0.05). The mRNA levels of S6 in the GL and GH-SB groups were downregulated compared with those in the FM group (*P* < 0.05). However, there were no significant differences in the mRNA levels of 3-PDK1, Akt, 4EBP1, EIF4E, EIF4B, Sin1, MHC I, and TCR mRNA in the FM, GL, GH, and GH-SB groups (*P* > 0.05).

### Distal Intestinal PI_3_K-Akt-mTOR Protein Expression

**Figure 5:**
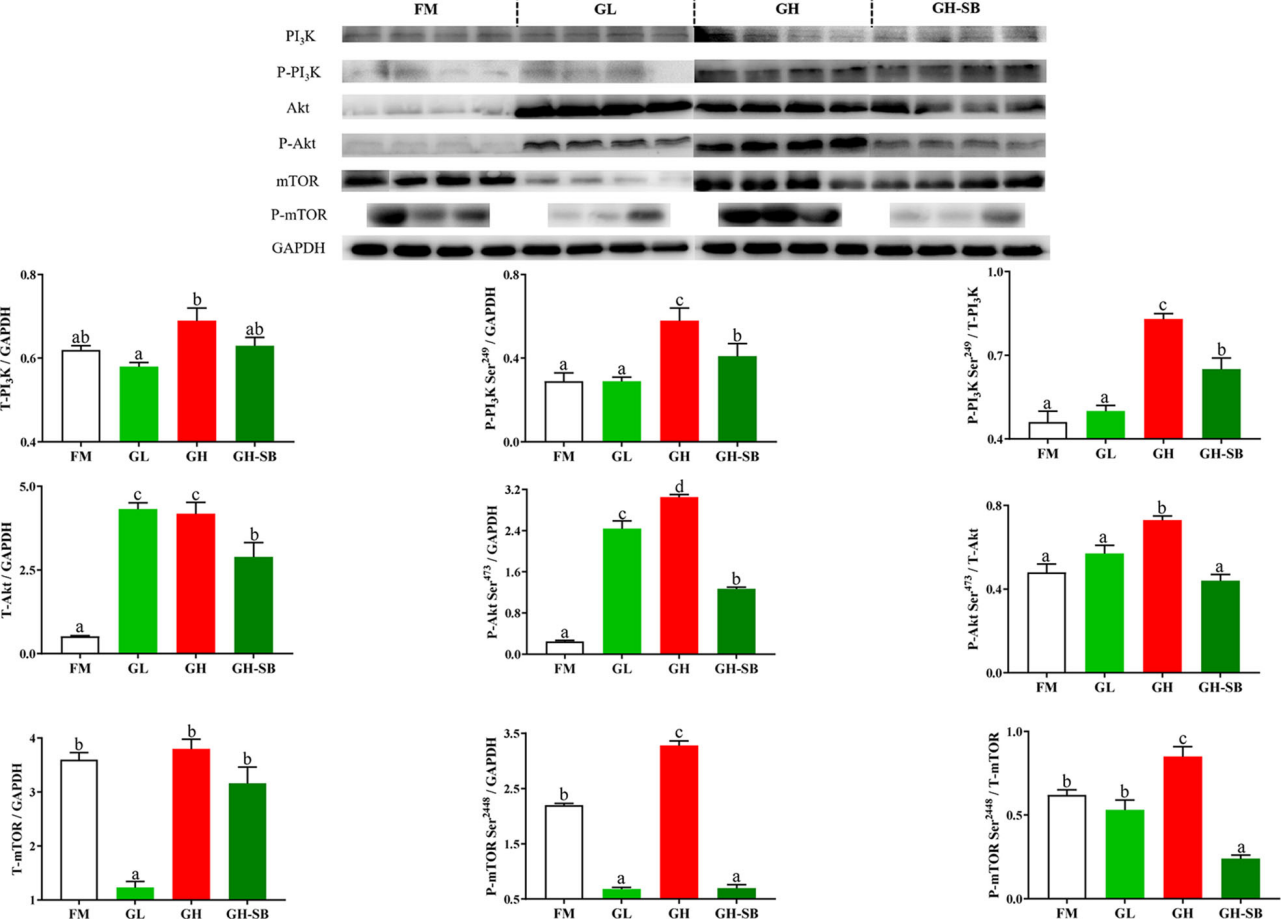
Dietary modulations of distal intestinal responses in the target of PI_3_K/Akt/mTOR signaling pathway. Results are represented as mean ± SE (n = 4). Significance was evaluated by one-way ANOVA followed by Tukey’s multiple range tests. FM, control diet; GL, containing 2% 11S diet, GH, containing 8% 11S diet, GH-SB, containing 8% 11S and 0.13% SB diet. ^a,b,c^Mean values among all treatments with different letters were significantly different when the interaction was significant (*P* < 0.05).

The distal intestinal PI_3_K/Akt/mTOR protein expression in DI is presented in **Figure 5**. The P-PI_3_K and P-PI_3_K/T-PI_3_K in the GH group showed a significant increase compared with those in the FM group (*P* < 0.05) but a significant decrease in the GH-SB group compared with those in the GH group (*P* < 0.05). By contrast, for T-PI_3_K, P-PI_3_K, and P-PI_3_K/T-PI_3_K, no significant differences were noted between the FM and GL groups. In addition, T-Akt and P-Akt levels in the GL and GH groups and P-Akt/T-Akt in the GH group were significantly increased compared with those in the FM group (*P* < 0.05). As for the GH-SB group, T-PI_3_K, P-PI_3_K, and P-PI_3_K/T-PI_3_K showed a significant decrease compared to those in the GL, GH, and GH groups, respectively (*P* < 0.05). T-mTOR and P-mTOR showed a significant decrease in the GL group as well as a significant decrease in the GH-SB group. P-mTOR and P-mTOR/T-mTOR increased significantly in the GH group (*P* < 0.05).

## Discussion

### Growth Performance and Challenge Test

11S is one of the antigen proteins with high content and strong antigenicity in soybean meal and its processed products. According to the protein structure, 11S generally accounts for about 40% of the total protein content of soybean meal (19). High-dose 11S destroys the morphological structure, reduces nutrient absorption, and reduces immunity of the intestine; however, low-dose 11S intake usually has an immune-enhancing effect. Li et al. showed that supplementing the feed with 3% of 11S increased the final weight and SGR of juvenile turbot, while 12% decreased weight and SGR (9). This outcome matches our results. In this experiment, growth performance in the GH group was significantly decreased by 8% 11S. This corresponds to trends found for SGR, feed intake (FI), and feed efficiency (FE) in grass carp (*Ctenopharyngodon idella*) and FE in Jian carp (*Cyprinus carpio* var Jian) (7), which were all also decreased by 8% 11S.

After adding sodium butyrate to the GH group to create group GH-SB, the WGR and SGR were significantly increased. The FCR was also significantly decreased compared with the GH group. Similar results were found in turbot (11) and rice field eel (*Monopterus albus*) (20). This indicates that sodium butyrate could alleviate growth inhibition caused by high levels of soybean meal or 11S in the feed. As an additive, SB can be absorbed in the intestinal lumen, and it can quickly provide energy for intestinal epithelial cells through oxidation (21). It is also an activator that improves intestinal villi proliferation and crypt deepening (22), and it enhances intestinal absorption capacity (13). Its special smell is very attractive to piglets, so it can be used as an attractant, SB also strengthens its immunity and digestion (23). These may be the reasons for the improved growth performance of the hybrid grouper.

To explore the effects of different doses of 11S and SB supplementation on the disease resistance of the hybrid grouper, we conducted a one-week challenge test experiment after the end of the 8-week breeding experiment. As a gram-negative bacterium, *Vibrio parahaemolyticus* has a wide distribution, mainly living in seawater, fish, shrimp, shells, and crustaceans (24). It usually causes gastrointestinal infections and extra-intestinal infections in aquatic animals. In addition, it can also cause symptoms such as diarrhea and fever if people eat raw or uncooked seafood with *Vibrio parahaemolyticus* (25). In gilthead sea bream (*Sparus aurata*) (26% replacement level) (26), the SGR was increased in the low soybean substitution level group, and similar results were found in Atlantic salmon (25% and 33% replacement levels) (27), white snook (*Centropomus viridis*) (30% replacement level) (28), and Russian sturgeon (*Acipenser gueldenstaedtii*) (20% replacement level) (29). This trend might be related to increased immunity. For this reason, we used *Vibrio parahaemolyticus* to conduct a challenge experiment to observe the change in cumulative mortality after the breeding experiment (**Figure S1**). We found that the CM in the FM and GL groups were not significantly different. In addition, no significant difference in CM was found between the GH and GH-SB groups. We speculated that a low dose of 11S and the addition of SB does not seem to be associated with disease resistance for the growth improvement of the hybrid grouper.

### Serum Immunity

IFN-γ is produced by Th1 and NK cells. Its main function is immune regulation, and it is an activator of phagocytes and neutrophils. It can promote the differentiation of T and B cells and enable various types of cells to express MHC class II antigens. In an inflammatory environment, iNKT cells are stimulated to produce IFN-γ. In this study, serum IFN-γ levels increased in the GL group and the GH group. This indicates that low-dose 11S could properly enhance immunity by increasing IFN-γ. When the amount of 11S increases, the secretion of IFN-γ will increase abnormally. Based on the results, this abnormal increase cannot be alleviated by supplementing with SB. IgG is the main antibody component in serum and extracellular fluid. It plays an important role in immunity against infections, especially in the secondary immune response (30). It is the main antibody of toxins, viruses, and bacteria, which can react with the corresponding antigen to eliminate the damage of the antigen to the body. In this experiment, after adding high-dose 11S, serum IgG showed a significant increase. This indicates that IgG is also involved in the 11S-mediated allergic reaction of the hybrid grouper, which is similar to the results of a study on piglets (31). After supplementing with SB, IgG was reduced to a certain extent, indicating that SB can alleviate the allergic reaction caused by 11S by lowering the serum IgG content. However, in nursery pigs, the content of IgG was upregulated by SB (32). Combined with the results of this experiment, we speculate that the role of SB mainly enhances serum immunity by modulating the secretion of host immune-enhancing cytokines in the hybrid grouper. This regulation could be closely related to the supplying of energy for intestinal epithelial cells and regulation of the expression of intestinal epithelial inflammatory factors (33).

IL-1β is mainly produced by mononuclear macrophages after being stimulated by external antigens which can activate T lymphocytes and B lymphocytes, thereby upregulating immune function (34). IL-1β is also an important indicator for measuring the severity of inflammatory bowel disease. The occurrence of enteritis is usually accompanied by an abnormal increase in serum IL-1β (35–37). In this experiment, supplementation with 11S at low and high levels reduced and increased the serum IL-1β content, respectively. After further supplementation with SB, the IL-1β content returned to the level of the control group. IL-1β is a pro-inflammatory cytokine with multiple biological activities. The reason for the increase of IL-1β in the GH group may be that when a high dose of 11S destroys small intestinal epithelial cells, a large number of monocytes in the intestinal mucosa propria are infiltrated, Th1 lymphoid immune cells are activated, and the pro-inflammatory factor IL is produced (38). In addition, IL-1β can stimulate T-type immune cells to differentiate and produce IFN-γ. In mice, SB may inhibit pro-inflammatory response by downregulating IL-1β (39). IL-1β may be decreased by SB for ischemic stroke in middle-aged female rats (40). These results are similar to the results of this experiment on hybrid grouper. Our results suggest that SB can also alleviate intestinal inflammation caused by high levels of 11S by inhibiting the secretion of IL-1β.

The massive production of TNF-α as a typical pro-inflammatory cytokine in humans is an important link in the pathogenesis of intestinal inflammation (41, 42). TNF-α is mainly produced by specific cells such as macrophages and monocytes, and it has a variety of biological activities. By mediating phosphorylation and ubiquitination of IkB, the nuclear localization sequence of NF-κB is exposed, and NF-kB is immediately transferred. It is located in the nucleus and binds to the NF-κB site to initiate gene transcription, leading to the release of large amounts of cytokines including TNF-α. The released cytokines can further activate NF-κB and intensify further inflammation (43). We found that high levels of 11S increased the serum TNF-α level of the hybrid grouper, indicating that 11S can induce intestinal inflammation by increasing TNF-α, which is similar to the results of studies on pigs (44) and Chinese mitten crabs (*Eriocheir sinensis*) (45). However, the aggravation caused by 11S in the hybrid grouper can be alleviated by supplementing with SB. We speculate that this may be related to the inhibitory effect of SB on NF-kB (46).

### Distal Intestinal Morphological Development (AB-PAS)

The fish intestine is the most important place for fish to absorb nutrients and is also an important immune organ. Whether the intestine is healthy and structurally intact directly determines the growth and immune performance of the fish (47). Compared with mammals, fish have thin intestinal walls and fragile intestines (only one-twentieth the thickness of the mammalian intestinal wall), which are extremely vulnerable to damage, destruction, and changes in external factors, especially feed ingredients. Therefore, repair and protection of fish intestines has always been one of the hot spots in fish nutrition and immunology research. The height of fish intestinal plica usually determines the fish’s immunity levels. Generally, the higher the fold, the stronger the immunity and absorption capacity; however, the fold height cannot accurately measure intestinal development. Therefore, we used the diameter of the intestine divided by the height of the plica to evaluate the histology of the intestine. Although there was no statistically significant difference between the Id/Ph of the GL group and the FM group in the distal intestine, there was still a certain degree of reduction. The GH group showed a significant increase, indicating that high doses of 11S can significantly inhibit intestinal development. This can be observed in the intestinal AB-PAS section (**Figure 2**). Adding 8% of 11S to the feed can significantly inhibit the plica height of the proximal, mid, and distal intestines of grass carp (7), which is a similar result to this experiment. In mice, the intestinal inflammation induced by SB also showed a decrease in plica height (48).

Furthermore, the muscle thickness of the distal intestine was also affected by 11S. The thickness of the intestinal muscle layer can reflect the peristaltic ability of the intestine, thereby preventing the occurrence of intestinal diseases to a certain extent (49). This shows that after adding a high dose of 11S to the feed, the peristalsis of the distal intestine is inhibited, while a low dose of 11S could promote peristalsis of the distal intestine. In the GH-SB group, we found that plica height, muscle thickness, and Id/Ph were all significantly improved compared with the GH group, indicating that SB can effectively alleviate the inhibition of development caused by high-dose 11S in the distal intestine of the hybrid grouper. SB can regulate the tight junction of the intestine (50) and enhance intestinal epithelial barrier function (51). We speculate that this positive protective effect on the intestines also exists in hybrid groupers. As previously mentioned, fish intestines have a strong immune function because they have a large number of mucous cells. They can produce acidic mucus, which plays an important role in protein digestion, bacterial capture, and immune stimulation (52). Studying the source of stimulation causing mucous cell differentiation, increased number and density, and improved immunity of fish is a valuable area of research.

From **Figure 2**, it can be found that type II mucous cells are mainly present in the distal intestine of the hybrid grouper (AB-PAS dyed blue). Interestingly, the density of mucous cells in the GL group increased significantly, suggesting that low doses of 11S can act as a source of stimulation to promote the differentiation of type II mucosal cells. This would cause secretion of acidic mucopolysaccharides and immunoglobulins with antibacterial and bactericidal effects, and enhancement of non-specific immunity in fish. The density of type II mucous cells in the GH group was significantly reduced, indicating that the high dose of 11S inhibited the differentiation of type II mucous cells. Type II mucosal cell density increased again after the addition of SB at high doses of 11S. This indicates that SB relieved the inhibition of type II mucosal cell differentiation caused by high doses of 11S, promoted differentiation, and performed normal physiological functions. This may be related to the ability of SB to induce cell differentiation (53) and promote anti-apoptosis (54).

### Distal Intestinal Immune Response

Changes in intestinal immune function are usually accompanied by the regulation of related immune pathways and the activation and inhibition of key components, which are finally manifested in growth and tissue morphology. Low-dose 11S has a certain immune enhancement effect on hybrid grouper, while high-dose 11S produces immunosuppression and induces enteritis. At the same time, the addition of sodium butyrate can act on the hindgut to make hybrid groupers exhibit better growth and immunity. However, it is currently unclear how this difference is regulated. A previous study showed that the uptake of soybean antigens by the intestine of aquatic animals mainly occurs in the latter half of the intestine of fish (55); therefore, we selected the distal intestine near the cloaca for a targeted study. To further explore how low and high doses of 11S and sodium butyrate regulate the distal intestine of the hybrid grouper, we performed the following quantitative research.

**Figure 6:**
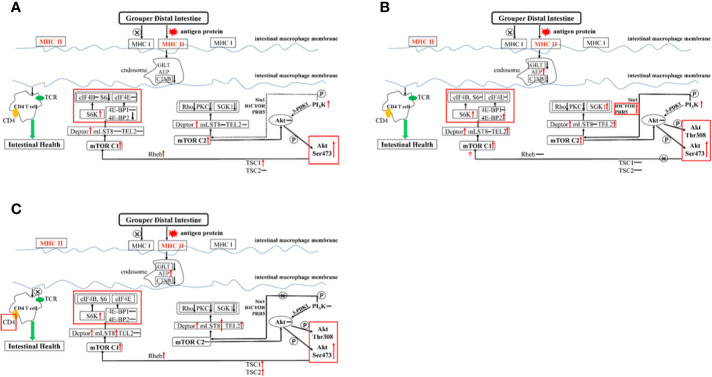
Summary of the potential pathways about exogenous antigen 11S processing and presentation in the distal intestine. Letter P in a circle, phosphorylation; ↓, down-regulated; ↑, up-regulated; –, constant; →, through; –⊗→, not through; ···, might through. **(A)** GL group; **(B)** BH group; **(C)** GSH-SB group.

The weight of glycinin molecules is 350 kDa. After the hybrid grouper ingests glycinin, most of the glycinin is degraded into peptides and amino acids. A small portion passes through the intestinal epithelium in the form of macromolecular proteins, completely enters the blood and lymph, and stimulates the intestinal mucosal immune tissue to produce related allergic reactions (56, 57). The major histocompatibility complex (MHC) is a highly polymorphic group of genes in vertebrate coding that is directly related to the immune response (58). Fish MHC molecules have abundant polymorphisms. The classic MHC I and MHC II molecules of bony fish belong to different linkage groups and play an important role in antigen presentation in the immune system (59). In this experiment, the addition of 11S at a low level (**Figure 6A**) showed no significant regulation in the expression of MHC I and MHC II, indicating that the normal immune recognition was not disturbed. The CTSB and AEP involved in the formation of endosomes were downregulated, which slowed the inhibition of intestinal cell differentiation by CTSB. Thus, AEP does not seem to be involved in this process. The PI_3_K protein family is involved in the regulation of cell proliferation, differentiation, apoptosis, and other cell functions. The activation of PI_3_K can produce the second messenger PIP3 on the plasma membrane (60). It can also act as a second messenger to bind to the PH domain of Akt, and make Akt Thr308 and Ser473 phosphoric acid under the catalysis of PDK1 and PDK2, respectively. As a highly conserved serine/threonine-protein kinase, mTOR usually forms two different complexes in the body, mTOR C1 and mTOR C2, which play an important role in regulating the intestinal immune response (61). PI_3_K RS5 was significantly upregulated, while Sin1, RICTOR, PRR5, and 3-PDK1, the four key components that act on the Akt pathway, did not change significantly, indicating that PI_3_K did not directly act on Akt or through mTOR C2. mTOR C2 was upregulated, and according to the results of the Western blot (**Figure 5**), Akt was phosphorylated at Ser473, indicating that mTOR C2 is involved in Akt activation. We found that both mTOR C1 and mTOR C2 were the Deptor parts which played a major regulatory role in the distal intestine of the hybrid grouper rather than mLST8 and TEL2. Deptor promoted the differentiation of intestinal cells by inhibiting the activity of Rho, PKC, and SGK1. Previous studies have found that Rho is highly expressed in the intestine of Crohn’s disease with intestinal inflammation (62). PKC induces keratinocyte apoptosis and intradermal inflammation through independent signaling pathways (63), and inflammation signals stimulate the expression of SGK1 in macrophages (64), indicating that low-dose 11S can inhibit inflammation by downregulating Rho, PKC, and SGK1. Similar to how Akt is phosphorylated at Ser473, TSC1 and TSC2 can also be phosphorylated to enrich Rheb, thereby activating mTOR C1, upregulating downstream S6K, and downregulating 4E-BP2 instead of 4E-BP1 through Deptor. 4E-BP2 can limit the anti-inflammatory response of macrophages by inhibiting IL-10 and cyclooxygenase-2 (65), and the activation of p70 S6K can reduce skin inflammation in atopic dermatitis (66). Therefore, in the hybrid grouper, low-dose 11S has a certain immune-enhancing effect, which may be closely related to inhibition of 4E-BP2 and promotion of the expression of p70 S6K.

When high-dose 11S was supplemented (**Figure 6B**), MHC I did not participate in regulation, but MHC II did, similar to the results of low-level addition. MHC II was activated, inhibiting CTSB and GILT. The key gene AEP in the process of CD4 T cell activation (67) was sharply upregulated, and the regulation of CD4 T cells was completely opened. From the results of CD4 glycoprotein and TCR, it is mainly the CD4 glycoprotein acting on the cell surface. PI_3_K was significantly upregulated, while Sin1 and 3-PDK1 still had no significant regulation. RICTOR and PRR5 were upregulated, indicating that PI_3_K does not directly act on Akt, but can activate mTOR C2 through RICTOR and PRR5 to further act on Akt. In contrast to the low dose, mTOR C2 also upregulates TEL2 while acting on Deptor. Deptor promotes the differentiation of intestinal cells by inhibiting the activity of Rho and PKC kinases, but not SGK1. At the same time, TEL2 activation leads to the activation of SGK1, leading to intensified intestinal inflammation. We found that in the step from Akt Ser473 phosphorylation to mTOR C1, TSC1 and TSC2 remained unchanged, and Rheb was not enriched. This result indicated that the path from Akt to mTOR C1 was blocked; however, mTOR C1 still showed high expression, indicating that there may be some other regulations. In a mouse intestinal gastric cancer model, mTOR C1 was overactivated, and the activation of the PI_3_K/mTOR C1 pathway was necessary for the occurrence of inflammation-related gastrointestinal tumors (68). Combined with the results of this experiment, the small molecule protein downstream of mTOR C1 seems to be more suitable as a marker to detect hybrid grouper enteritis. The two key assembly factors, Deptor and TEL2, were simultaneously activated, and they both upregulated S6K and 4E-BP2, reducing distal intestine anti-inflammatory ability.

SB has a positive effect on hybrid groupers when considering growth, serum indexes, and distal intestinal development. To explore how SB protects the distal intestine, thereby alleviating the intestinal damage caused by high doses of 11S, we further supplemented SB to the GH group. After high-dose 11S supplementation with SB (**Figure 6C**), we found that MHC I and MHC II were similar to the GL group. Neither were significantly upregulated and both were in a normal state. Compared with the GH group, MHC II was significantly downregulated. Similar to the high-dose 11S group, CTSB and GITL were inhibited, AEP was still sharply upregulated, and the regulation of CD4 T cells was activated. The difference is that, from the expression of CD4, the glycoprotein on the surface of CD4 T cells seems to stop receiving signals from the endosomes. PI_3_K phosphorylation increased in the GH-SB group compared with that in the FM group (**Figure 5**). Sin1, RICTOR, PRR5, and 3-PDK1 did not cause significant regulation and mTOR C2 was not activated, indicating that this pathway was blocked to a certain extent. Further research found that, compared with the high-dose group, mLST8 was upregulated and SGK1 was thereby inhibited, which protected the distal intestine. After Akt Ser473 is phosphorylated to mTOR C1, both TSC1 and TSC2 are activated, which causes Rheb enrichment and activation of mTOR C1. mTOR C1 upregulates S6K and acts on Deptor and mLST8 simultaneously, but 4E-BP1 and 4E-BP2 remain unchanged to protect CD4 T cells from normal differentiation and function.

## Conclusions

In the current study, low-dose 11S increased the SGR, feed utilization rate, and density of distal intestinal-type II mucous cells of the hybrid grouper. The serum immune function was enhanced by the secretion of IFN-γ. High-dose 11S hurt SGR, feed utilization, and distal intestinal development. The serum immune function was weakened due to the abnormal increase in IgG and IL-1β. After supplementation with sodium butyrate, IgG, IL-1β, and TNF-α can be adjusted to normal levels, causing enhancement to immunity in the distal intestine. Hybrid groupers present foreign antigen proteins through MHC II. PI_3_K is not associated with mTOR C2. mTOR C1 improves intestinal immunity by inhibiting 4E-BP2 under low-dose 11S. PI_3_K associated with mTOR C2 upregulates SGK1 through RICTOR and PRR5, while mTOR C1 upregulates 4E-BP2 through TEL2, aggravating intestinal inflammation. After supplementing with SB, PI_3_K/mTOR C2 is blocked, and mLST8 inhibits SGK1. At the same time, mTOR C1 inhibits the expression of 4E-BP2 through mLST8 and relieves the inflammation caused by the high dose of 11S in the intestine. In summary, the hybrid grouper obtained different serum and intestinal immune responses under the conditions of different doses of 11S, and these responses were ultimately manifested in growth performance. Sodium butyrate can effectively improve serum immunity and relieve intestinal inflammation caused by high dose 11S.

## Data Availability Statement

The raw data supporting the conclusions of this article will be made available by the authors, without undue reservation.

## Ethics Statement

The animal protocol was approved by the ethics review board of Guangdong Ocean University. All procedures were performed according to the standards of the National Institutes of Health Guide for the Care and Use of Laboratory Animals (NIH Publication No. 8023, revised 1978) and relevant Chinese policies.

## Author Contributions

All authors have actively contributed to this study. The author’s contributions are as follows: HL and BT designed the study. BY conducted the study and analyzed the data. XD participated in the interpretation of the results. BY wrote the manuscript. QY, SC, and SZ purchased the reagent supplies. HL revised the manuscript. All authors contributed to the article and approved the submitted version.

## Funding

This work was supported by the National Key R&D Program of China (2019YFD0900200), the National Natural Science Foundation of China (no. 31772864), and the Natural Science Foundation of Guangdong Province (2018A030313154&2020A1515011129).

## Conflict of Interest

The authors declare that the research was conducted in the absence of any commercial or financial relationships that could be construed as a potential conflict of interest.
