# Supplementary material for: MHC II-PI3K/Akt/mTOR Signaling Pathway Regulates Intestinal Immune Response Induced by Soy Glycinin in Hybrid Grouper: Protective Effects of Sodium Butyrate
Source: Front Immunol. 2021 Jan 18;11:615980. doi: 10.3389/fimmu.2020.615980 (PMC7849651; doi:10.3389/fimmu.2020.615980)
Supplement: Supplementary file 1 [file Table_1.docx]

Supplementary Material

**Figure. S1** Cumulative mortality (%) for four treatments against *Vibrio parahaemolyticus* of juvenile hybrid grouper (*Epinephelus fuscoguttatus*♀*×E. lanceolatus*♂) fed the experimental diets for 8 weeks.

**Table. S1** Composition of the diets

|  | Diets | | | |
| --- | --- | --- | --- | --- |
| Ingredients/% | FM | GL | GH | GH-SB |
| Red fishmeal | 40.00 | 40.00 | 40.00 | 40.00 |
| Casein | 11.54 | 10.09 | 5.72 | 5.72 |
| Gelatin | 2.89 | 2.52 | 1.43 | 1.43 |
| Wheat flour | 20.00 | 20.00 | 20.00 | 20.00 |
| Fish oil | 4.72 | 4.72 | 4.72 | 4.72 |
| Soy lecithin | 2.00 | 2.00 | 2.00 | 2.00 |
| Calcium monophosphate | 1.00 | 1.00 | 1.00 | 1.00 |
| ^a^Vitamin premix | 0.20 | 0.20 | 0.20 | 0.20 |
| ^b^Mineral premix | 0.50 | 0.50 | 0.50 | 0.50 |
| Antioxidants | 0.05 | 0.05 | 0.05 | 0.05 |
| Choline chloride | 0.50 | 0.50 | 0.50 | 0.50 |
| Glycinin 11S | 0 | 2.00 | 8.00 | 8.00 |
| Vitamin C | 0.05 | 0.05 | 0.05 | 0.05 |
| Cellulose microcrystalline | 16.55 | 16.33 | 15.68 | 15.55 |
| ^c^Methionine | 0 | 0.02 | 0.08 | 0.08 |
| ^c^Lysine | 0 | 0.02 | 0.07 | 0.07 |
| Microencapsulated SB | 0 | 0 | 0 | 0.13 |
| Proximate composition (% air dry matter) | | | | |
| ^d^Crude protein | 47.56 | 47.67 | 47.72 | 47.67 |
| ^d^Crude lipid | 10.55 | 10.23 | 10.23 | 10.40 |
| ^d^Moisture | 9.08 | 9.01 | 9.07 | 9.04 |

^a^Vitamin premix (g/kg mixture): vitamin B1, 17.00 g; vitamin B2, 16.67 g; vitamin B6, 33.33 g; vitamin B12, 0.07 g; vitamin K, 3.33 g; vitamin E, 66.00 g; retinyl acetate, 6.67 g; VD, 33.33 g, nicotinic acid, 67.33 g; D-calcium pantothenate, 40.67 g; biotin, 16.67; folic acid, 4.17 g; inositol, 102.04 g; cellulose, 592.72 g. All ingredients were diluted with corn starch to 1 kg.

^b^Mineral premix (mg g-1 mixture):CaCO_3_, 350 g; NaH_2_PO_4_·H_2_O, 200 g; KH_2_PO_4_, 200 g; NaCl, 12 g; MgSO_4_·7H_2_O, 10; FeSO_4_·7H_2_O, 2 g; MnSO_4_·7H_2_O, 2 g; AlCl_3_·6H_2_O, 1 g; CuCl_2_·2H_2_O, 1 g; KF, 1 g; NaMoO_4_·2H_2_O, 0.5 g; NaSeO_3_, 0.4 g; CoCl_2_·6H_2_O, 0.1 g; KI, 0.1g; zeolite powder, 219.9 g. All ingredients were diluted with corn starch to 1 kg. (Obtained from Zhanjiang Yuehua Feed Co. Ltd. , Zhanjiang, China).

^c^Methionine and lysine were added to balance amino acid with control group.

^d^Crude protein, crude lipid and moisture contents were measured value.

**Table. S2** Essential amino acid profile (%) of the diets used in the experiment.

| Essential amino acids | Diets | | | |
| --- | --- | --- | --- | --- |
|  | FM | GL | GH | GH-SB |
| Methionine | 1.02 | 0.94 | 1.12 | 1.06 |
| Lysine | 3.12 | 3.03 | 3.20 | 3.16 |
| Threonine | 1.77 | 1.78 | 1.86 | 1.81 |
| Isoleucine | 1.78 | 1.80 | 1.95 | 1.88 |
| Histidine | 1.31 | 1.34 | 1.38 | 1.35 |
| Valine | 2.12 | 2.08 | 2.25 | 2.17 |
| Leucine | 3.21 | 3.27 | 3.30 | 3.26 |
| Arginine | 2.28 | 2.25 | 2.73 | 2.69 |
| Phenylalanine | 1.84 | 1.79 | 2.00 | 1.95 |
| Tyrosine | 1.28 | 1.19 | 1.43 | 1.37 |
| Aspartic acid | 3.63 | 3.62 | 4.12 | 4.08 |
| Serine | 1.82 | 1.91 | 1.88 | 1.90 |
| Glutamate | 7.20 | 7.39 | 7.74 | 7.60 |
| Glycine | 2.65 | 2.61 | 2.58 | 2.55 |
| Alanine | 2.42 | 2.42 | 2.20 | 2.18 |
| Cystine | 0.42 | 0.42 | 0.54 | 0.44 |
| Proline | 2.95 | 3.16 | 2.65 | 2.67 |

**Table. S3** Primers sequences used for real-time quantitative PCR.

| Name | Sequence (5’-3’) | Product size (bp) |
| --- | --- | --- |
| PI_3_K RS5 | GCCGAGGAGGAAGAGGATGTAGAC | 188 |
| 3-PDK1 | GGCAGCCATTACTGGAGCTTCTC | 137 |
| TSC1 | GTGACGCCGCTGGTTGAAGAC | 189 |
| TSC2 | TACGGAGACGACGGAGAGTTCAC | 80 |
| Akt | GGCAGGATGTGGTACAGAAGAAGC | 123 |
| Ikkα | GTACCTCCTAAGGATTGCCTGTGC | 195 |
| Rheb | CCATGATGTAGAGCCGCCCA | 87 |
| Raptor | GGTCAAGGTCAACGAGGATCACTG | 194 |
| PRAS40 | CTGACGGCGAGGAGGAGAGC | 99 |
| mTOR | TGAGGAGTGGACGCTGGTGAG | 193 |
| mTOR C1 | GGTCAAGGTCAACGAGGATCACTG | 194 |
| mTOR C2 | CCACACTCAGCCAACACTACTTCC | 139 |
| Deptor | CATCATCCAGCACGGTGAGGAAG | 146 |
| mLST8 | ATCAACTGTGTCTGCCTGCATCC | 104 |
| TEL2 | ACCTGCTGCTGGACCTCAGTG | 169 |
| 4E-BP1 | CGGATAGCAGCAACACTGGTGAAG | 155 |
| 4E-BP2 | GTCTTCCAGTCGTCAGCATAGCG | 82 |
| p70 S6K | CCGCAACGCCAAGGACACAG | 141 |
| EIF4E | CGTCTGTGGAGCTGTGGTCAAC | 159 |
| EIF4B | GGATGGTAGCAGCGGCAACG | 142 |
| S6 | TGCCAATCTCAGCGTGCTCAAC | 158 |
| Sin1 | GTCCACGACCTTATTGGCCTCATC | 121 |
| RICTOR | CCACACTCAGCCAACACTACTTCC | 139 |
| PRR5(p) | CGCAGCAGAGACAGCAGCAG | 121 |
| RhoA | CCTGCGTAACGATGAGCACACC | 99 |
| PKC | CCACTCACATAAGGCAGCGTAGAC | 193 |
| SGK1 | ACAGGACGGTAGACTGGTGGTG | 118 |
| CIITA | GTTGTCCTGGTGTTGAAGCCTCTC | 161 |
| RFX5 | TGGACTGTGGTAGGAAGCTGGAG | 135 |
| CREB1 | CCGTAGACAGCGTGACAGATTGC | 137 |
| NFY | ACCACTGCCACCACCACTACTG | 105 |
| MHCI | CGCACGATAGGATCAGGCAAGTC | 171 |
| MHCII | CAAGATTGCCATCGGAGCCTCAG | 131 |
| GILT | GCCAGCATGGACCACCAGAATG | 80 |
| AEP | TGCTGACAAGACTGCCATCACAC | 107 |
| CTSB | TGATCTGTTGCAAGGTCTCGTTCC | 100 |
| TCR | GCTTGGCCTCGCTTCTTCTTCC | 119 |
| CD4 | GCTGCCTTGACTGTACGGACTG | 165 |
| β-actin | TACGAGCTGCCTGACGGACA | 239 |
